# Differences in Weight Loss Postsleeve Gastrectomy Among Patients With Various Types of Obesity Based on Waist-To-Hip Ratio Classification

**DOI:** 10.1155/jobe/4236484

**Published:** 2025-03-13

**Authors:** Pengxiang Luan, Yunmiao Pan, Sanyuan Hu, Mingwei Zhong

**Affiliations:** ^1^Department of General Surgery, Shandong Provincial Qianfoshan Hospital, Cheeloo College of Medicine, Shandong University, Jinan, Shandong, China; ^2^Department of General Surgery, Qilu Hospital, Cheeloo College of Medicine, Shandong University, Jinan, Shandong, China; ^3^Department of General Surgery, The First Affiliated Hospital of Shandong First Medical University, Shandong Provincial Qianfoshan Hospital, Jinan, Shandong, China

**Keywords:** bariatric surgery, laparoscopic sleeve gastrectomy, obesity, waist-to-hip ratio, weight loss

## Abstract

**Background:** In recent years, laparoscopic sleeve gastrectomy (LSG) has become the main surgical procedure for weight loss, and most clinical studies have focused on the postoperative complications and treatment of metabolic syndrome after LSG. However, it is not clear whether there is a difference in the postoperative weight loss effect between patients with central and noncentral obesity after LSG.

**Purpose:** To investigate the effect of LSG on weight loss in patients with central obesity and relationship between preoperative waist–hip ratio and weight loss effect.

**Methods:** We conducted a retrospective study comprising 360 patients who underwent LSG at the Qianfoshan Hospital, Jinan, Shandong Province, China, between 2019 and 2024. Based on the preoperative waist-to-hip ratio (WHR), the participants were divided into central and noncentral obesity groups, and various quantitative and preoperative biochemical indices were measured. Most patients were followed up for at least 6 months.

**Results:** There were significant differences in weight loss outcomes between women in the central and noncentral obesity groups in the first and third months after surgery; however, no significant differences were observed in the sixth and twelfth months. No significant differences were observed in weight loss outcomes between men in the central and noncentral obesity groups. There were significant differences in the development of central obesity between the two sexes and between those with and without type 2 diabetes. There were significant differences in body mass index (BMI) and white blood cell counts between women in the central and noncentral obesity groups, with patients with central obesity having higher BMI values and white blood cell counts before surgery. There were significant differences in the platelet count (PLT), gamma-glutamyl transferase (GGT), glycosylated hemoglobin A1c (HbA1c), and fasting plasma glucose (FPG) levels between men in the central and noncentral obesity groups, with patients with central obesity having lower PLT, higher GGT, HbA1c, and FPG levels. There was a significant correlation between WHR and early weight loss outcomes after surgery.

## 1. Introduction

Obesity has become a major public health problem in most developed countries, with the number of obese adults worldwide reaching 500 million and growing rapidly [1, 2]. Obesity not only increases the risk of cardiovascular, digestive, and endocrine system diseases but also seriously damages human health [3–6]. According to the latest international guidelines for metabolic and bariatric surgery (MBS) indications, MBS is recommended for individuals with a body mass index (BMI) > 35 kg/m^2^ regardless of the presence or severity of comorbidities. For individuals with a BMI valve ranging from 30 to 34.9 kg/m^2^ and metabolic diseases, MBS treatment should be considered. In addition, the BMI threshold should be adjusted for Asian populations: BMI > 25 kg/m^2^ indicates clinical obesity, while BMI > 27.5 kg/m^2^ should be considered for MBS treatment [7]. Based on the latest guidelines for MBS indications, Asian individuals with a BMI > 27.5 kg/m^2^ are recommended to undergo MBS treatment. BMI is commonly used as a measure of total body fat content; however, the waist-to-hip ratio (WHR) can provide a more intuitive and straightforward reflection of fat distribution in various types of patients with obesity. Relevant studies indicate that the distribution of adipose tissue plays a significant role in the development of obesity [8]. Patients with more abdominal fat are more likely to suffer from obesity-related complications. Therefore, the relationship between abdominal fat and obesity is very close. According to a 2018 survey undertaken by the International Federation for the Study of Obesity (IFSO), laparoscopic sleeve gastrectomy (LSG) has become the most common weight-loss surgery globally, accounting for 55.4% of all weight loss surgeries [9, 10]. However, it is unclear whether LSG has different effects on weight loss in various types of patients with morbid obesity.

## 2. Materials and Methods

### 2.1. Participants

This study included 360 patients with obesity who underwent LSG at the Qianfoshan Hospital, Jinan, Shandong Province, China, from December 2019 to September 2024. All patients received unified postoperative guidance and health education. This study included 109 men and 251 women. The mean BMI value was 41.25 (30.12–69.44) and the mean age was 32 years (14–62).

### 2.2. Inclusion/Exclusion Criteria

Inclusion criteria were as follows: (1) age 16–65 years old; (2) patients who met the surgical indications of the American Society for Metabolic and Bariatric Surgery and IFSO MBS indications (2022 edition); and (3) postoperative patients who could be followed up normally.

Exclusion criteria were as follows: (1) patients who needed to use obesogenic drugs due to their condition after surgery; (2) patients who became pregnant shortly after surgery; and (3) patients lost to follow-up for unknown reasons.

### 2.3. Grouping Method

Central obesity was defined as a WHR > 0.85 in women and > 1.0 in men. Noncentral obesity was defined as a WHR ≤ 0.85 in women and ≤ 1.0 in men [11, 12].

### 2.4. Data Collection

Data were collected independently by two individuals. Weight was measured without shoes and with minimal clothing to an accuracy of 0.1 kg. The height measurements were accurate to 0.01 m. BMI was calculated as the weight in kilograms divided by the square of the height in meters. The World Health Organization (WHO) Stepwise Approach to Surveillance protocol for measuring waist circumference instructs that the measurement be made at the approximate midpoint between the lower margin of the last palpable rib and the top of the iliac crest. The hip circumference should be measured around the widest part of the hip. Measurements were made using antitensile tape, which was placed close to the body of the patient. Participants should stand with their feet together, arms at their sides, weight evenly distributed, and wear a minimal amount of clothing. Participants should be relaxed and measurements should be taken at the end of normal expiration. Each measurement should be repeated twice. If the two measurements are within 1 cm of each other, the mean value should be calculated. If the difference between the two measurements is more than 1 cm, the two measurements should be repeated [11]. After LSG, we conducted thorough follow-ups at 1, 3, 6, and 12 month postsurgery through a combination of hospital visits and telephone interviews. These follow-up visits included assessments of postoperative height and weight, procedure-related complications, and the remission of preoperative comorbidities.

Per cent total weight loss (% TWL) was calculated using the following formula:

(1) $$\begin{array}{c} \frac{\text{Weight loss}}{\left(\text{preoperative weight}\right)}\times 100\%. \end{array}$$

Per cent excess weight loss (% EWL) was defined as follows:

(2) $$\begin{array}{c} \%\text{EWL}=\left(\frac{\text{weight loss}}{\text{baseline excess weight}}\right)\times 100\%. \end{array}$$

Baseline excess weight was calculated as follows:

(3) $$\begin{array}{c} \text{Baseline excess weight}=\text{baseline weight}—\text{minus ideal weight}. \end{array}$$

The ideal weight is based on the weight of the person at a BMI of 23 kg/m2 [13].

### 2.5. Laboratory Measurements

Fasting (12 h fast) blood samples were obtained for blood chemistry, including blood cell analysis (5 class method), albumin, glucose, triglycerides, high-density lipoprotein, low-density lipoprotein, cholesterol, alanine aminotransferase, aspartate aminotransferase, glycosylated hemoglobin A1c, fasting insulin, and fasting C-peptide.

### 2.6. Statistical Analysis

The study population was analyzed overall and then, after grouping according to sex, for men and women. Central obesity was classified according to WHR. Descriptive data satisfying normal distribution were expressed as the mean ± standard deviation and those satisfying skewed distribution were expressed as the median (P25 and P75). The incidence of obesity-related complications was compared between the central and the noncentral obesity groups. The chi-square test was used for preoperative evaluation. Two independent sample *t*-test and rank sum test were used to compare the characteristics between the two groups, and the *t*-test and rank sum test were used to compare the difference of weight loss effect. Linear regression was used to determine the factors affecting EWL and TWL. A two-tailed *p* value < 0.05 was used to infer statistical significance. Statistical analysis was performed using SPSS software Version 26.0 for Windows (SPSS, Inc., Chicago, IL, United States).

## 3. Results

### 3.1. Baseline Patient Characteristics

A total of 360 patients were enrolled in this observational study (men: 109, 30.3%; women: 251, 69.7%). The mean BMI of all patients was 41.25 (30.12–69.44), and the mean age was 32 years (14–62). The mean BMI of the men was 45.57 ± 7.74, and the mean waist-hip ratio was 1.01 ± 0.05. The mean BMI of the women was 38.89 (35.20, 42.32), and the mean WHR was 0.92 ± 0.07. A total of 360 patients completed the 1 month follow-up, 312 patients completed the 3 month follow-up, 208 patients completed the 6 month follow-up and 136 patients completed the 12 month follow-up.

### 3.2. Comparison of Preoperative Information

In obese patients, various obesity-related comorbidities are common. Women and patients with type 2 diabetes are more likely to develop central obesity (Table 1). There were significant differences in BMI and white blood cell counts between women in the central and noncentral obesity groups. Women with central obesity had higher preoperative BMI and white blood cell counts (Table 2). The differences in GGT, PLT, HbA1c, and FPG levels were more obvious in men of the central and noncentral obesity groups. Men with central obesity had lower PLT and higher GGT, HbA1c, and FPG levels (Table 3).

### 3.3. Weight Loss

**Figure 1:**
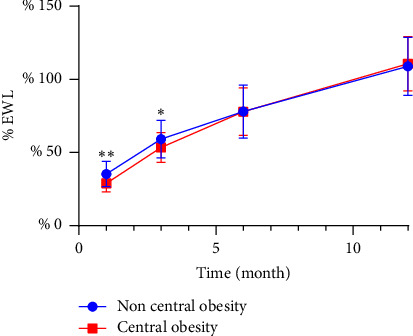
Excess weight loss (% EWL) after sleeve gastrectomy in women. ^∗∗^refers to a *p* value less than 0.01, ^∗^refers to a *p* value less than 0.05.

**Figure 2:**
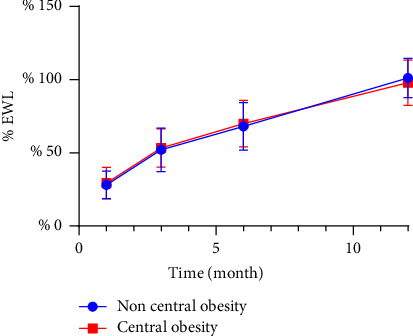
Excess weight loss (% EWL) after sleeve gastrectomy in men.

When EWL was used as a measure of postoperative weight loss effect, women in the noncentral obesity group showed a better weight loss effect than those in the central obesity group in the first and third months after surgery, while there was no significant difference in the sixth- and twelfth-months postsurgery (Table 4 and Figure 1). There was no difference in postoperative weight loss between men in the central and noncentral obesity groups of males (Table 5 and Figure 2).

**Figure 3:**
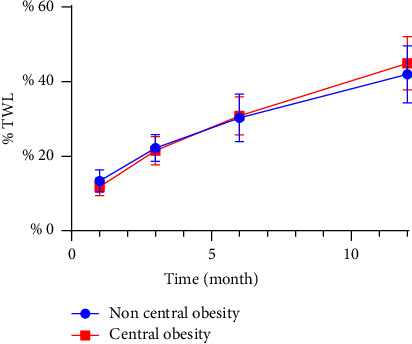
Total weight loss (% TWL) after sleeve gastrectomy in women.

**Figure 4:**
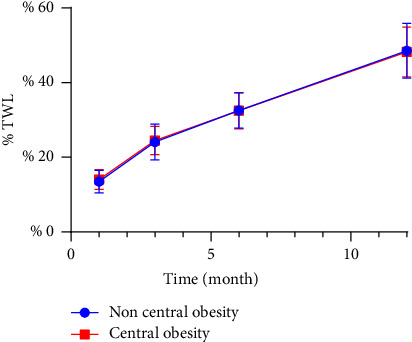
Total weight loss (% TWL) after sleeve gastrectomy in men.

When TWL was used as a measure of postoperative weight loss effect, the effect of weight loss in the first month after surgery in women with noncentral obesity was slightly better than that in women with central obesity. There was no difference between the third, sixth, and twelfth months after surgery (Table 4 and Figure 3). There was no difference in postoperative weight loss between the men in the central and noncentral obesity groups (Table 5 and Figure 4).

We carried out multiple linear regression analysis to further explore the factors affecting the weight loss effect. To evaluate the influence of central obesity, we conducted a linear regression analysis of % TWL and % EWL at each time point after surgery in Table 6 and 7. % TWL, % EWL, and BMI were approximately normally distributed. There was no significant multicollinearity for the factors affecting % TWL and % EWL. We observed that WHR significantly affected weight loss in the first and third postoperative months. The effect of BMI on weight loss within 1 year after surgery was statistically significant. Patient gender and age demonstrate no significant association with postoperative weight loss outcomes. Similarly, preoperative comorbidities such as metabolic syndrome or type 2 diabetes mellitus do not exhibit clinically meaningful effects on weight reduction efficacy following surgery. However, the temporal impact of pre-existing metabolic disorders on postoperative weight loss varies significantly: a history of hypertension is associated with attenuated weight loss during the first postoperative month, while hyperlipidemia predominantly influences outcomes in the third postoperative month. The research findings demonstrate that patients with central obesity complicated by hypertension and/or hyperlipidemia exhibit significantly restricted weight loss during the early postoperative period. These findings underscore the importance of considering specific metabolic comorbidities when formulating postoperative management strategies for this patient population.

## 4. Discussion

Recently, many studies have used computed tomography scans to quantify the abdominal fat area to analyze obesity and related comorbidities [14]. Therefore, we considered an approach that uses anthropometric parameters to determine fat distribution in patients with obesity to analyze differences in postoperative weight loss. WHR can concisely and clearly indicate the fat distribution of patients, and it is not harmful to patients. On the basis of WHR, patients could be classified as having central or noncentral obesity according to the WHO criteria [11]. Given that the participants in this study were all Chinese patients, a WHR cutoff point suitable for Asian patients with obesity was used [12].

This study investigated the difference in weight loss in patients with various types of obesity after LSG. The number of women in our cohort was twice that of men, but the mean BMI of men was higher than that of women. We found that there was a significant difference in the weight loss effect exhibited by women in the central and noncentral obesity groups in the early postoperative period, but there was no significant difference in the weight loss effect of the men in these two patient groups. This may be related to the different metabolic characteristics and physiological structure of men and women, which needs further study. We conducted an analysis of weight loss effect according to sex because the cutoff points for WHR differed between men and women and because women were more likely to have central obesity (Table 2). Based on our data, we suggest that reasonable diet and exercise interventions should be carried out for women with central obesity in order to obtain better weight loss in the early postoperative period.

The results were different when we employed various other indicators (such as % EWL or % TWL) to measure the effect of postoperative weight loss. % EWL is more focused on the loss of excess weight and can more accurately reflect the success of surgery, especially for those who are already overweight but were unable to reach the ideal weight [15]. % TWL reflects the overall weight loss after surgery [15]. Many studies have found that there are significant differences in % EWL, but not in % TWL, due to the differences in regions, populations, and types of surgery [16]. In addition, some data were missing from each phase of our postoperative follow-up data, which may have had some effect on the results.

In the present study, women with central obesity had higher BMI and white blood cell counts, which were associated with more abdominal fat in patients with central obesity. Abdominal adipose tissue secretes a variety of inflammatory factors, such as tumor necrosis factor and interleukin-6, which can stimulate the immune system and lead to leukocytosis [17]. Men with central obesity had higher HbA1c and FPG levels, which is consistent with the fact that patients with type 2 diabetes are more likely to develop central obesity. Previous studies have shown that patients with type 2 diabetes mellitus and central obesity are at a greater risk of cardiovascular disease [18].

Central obesity represents more abdominal fat, and increased abdominal fat not only affects sleep quality but also increases the risk of gout [19, 20]. Studies have shown that patients with central obesity are also more likely to suffer from cholelithiasis, and WHR is positively correlated with the severity of hepatic steatosis [21, 22]. WHR was found to be the best predictor of hypertension among 10 obesity-related indicators [23]. Therefore, central obesity is closely related to a variety of obesity-related complications. Hence, it is necessary to carry out reasonable dietary and exercise interventions and even drug intervention for patients with central obesity after surgery.

One study found no association between WHR and percent weight change with diet management alone for weight loss [24]. Therefore, the use of LSG in the treatment of central obesity is highly effective.

This study primarily focuses on the waist-to-weight ratio as a key predictive indicator for postoperative weight loss outcomes. However, it is important to note that other multidimensional factors may also influence weight loss efficacy, including demographic characteristics (gender and age), clinical features (preoperative comorbidities), behavioral factors (physical activity levels and dietary adherence), and genetic factors (genetic predisposition) [25–28]. The research findings demonstrate that patients with central obesity complicated by hypertension and/or hyperlipidemia exhibit significantly restricted weight loss during the early postoperative period. For this specific population, implementing early dietary interventions and exercise guidance can significantly enhance weight loss outcomes.

However, this study has the following limitations: First, postoperative dietary management and exercise interventions were not included as study variables. The existing literature suggests that systematic lifestyle interventions can lead to more significant weight loss outcomes in the year after surgery [25]. Second, the influence of genetic factors was not considered. Research indicates that patients carrying multiple obesity-related genetic loci tend to have relatively poorer weight loss outcomes after surgery [27, 29].

Based on these findings, future research will focus on the following areas for improvement: (1) developing a multifactorial predictive model that incorporates genetic predisposition; (2) systematically evaluating the long-term effects of postoperative lifestyle interventions; and (3) exploring the mechanisms of gene-environment interactions on weight loss efficacy. Delving deeper into these research directions will contribute to the development of personalized postoperative management strategies.

The follow-up period in this study was limited to 12 months postoperatively, which may not sufficiently reflect the long-term stability of weight loss outcomes or comprehensively assess potential long-term complications (such as weight regain and micronutrient deficiencies). Based on existing literature, the weight stabilization period for patients after laparoscopic sleeve gastrectomy (LSG) typically occurs beyond 12 months postoperatively, suggesting that extending the follow-up duration holds significant clinical value for phenotype-specific analysis [30]. Therefore, we recommend that future research prioritize the establishment of longitudinal follow-up cohorts spanning 3–5 years. Such a study design would facilitate the evaluation of the durability of weight loss outcomes in patients with different obesity types. However, due to the current lack of systematic long-term follow-up data, these research directions have yet to be realized. This limitation underscores the importance of establishing standardized long-term follow-up mechanisms, which will be a key focus of our team's next research efforts.

Our study has several limitations: (1) This study utilized a retrospective design, which inherently limits the ability to establish causal relationships between preoperative WHR classification and postoperative weight loss outcomes. Potential confounding factors, such as unmeasured lifestyle variables or selection bias, may influence the observed associations. Future prospective studies with standardized protocols for data collection and adjustment for confounding variables (e.g., socioeconomic status and medication use) are needed to validate these findings. (2) The differences in preoperative laboratory examination data between men and women cohorts may be related to insufficient sample size, and the fact that long-term follow-up data are missing, which may have a certain impact on the analysis of the difference of weight loss effect. (3) The absence of data on postoperative behavioral changes (such as dietary and exercise habits) and genetic factors represents a significant limitation. These variables should be prioritized in future research directions. (4) The follow-up period was only 1 year, so the long-term effect of weight loss could not be evaluated. Future research should focus on this aspect. (5) This study only focused on the Chinese population and was a single-center study. Due to the differences in WHR cutoff points in different countries and regions, future multicenter and multiregion studies should be carried out to clarify the difference in weight loss effect between the central and non-central obesity groups.

## 5. Conclusion

LSG can effectively treat morbid obesity and alleviate the related metabolic diseases associated with obesity, as well as reduce postoperative complications. Women with central obesity have worse early weight loss outcomes. The WHR can serve as an independent predictor of early weight loss outcomes. This can provide corresponding postoperative treatment plans for patients with different types of obesity to achieve better weight loss outcomes.

## Figures and Tables

**Table 1:** Correlation of central obesity with sex and various diseases.

Variables	Noncentral obesity	Central obesity	*X* ^2^/*t*/*Z*	*p* value
Sex (women)	43	208	38.542	< 0.001^c^
Metabolic syndrome	32	108	1.700	0.192
Hypertension	35	84	0.685	0.408
T2DM	30	114	4.176	0.041^a^
Hyperlipidemia	34	111	1.286	0.257
OSAHS	64	152	2.467	0.116

*Note: N* = 360.

Abbreviations: OSAHS, obstructive sleep apnea–hypopnea syndrome; T2DM, type 2 diabetes mellitus.

^a^ *p* < 0.05.

^c^ *p* < 0.001.

**Table 2:** Correlation of preoperative clinical indicators between the two populations of women.

Variables	Noncentral obesity	Central obesity	*X* ^2^/*t*/*Z*	*p* value
Number	43	208		
Age (years)	29 (26, 35)	32 (26.25, 37)	−1.127	0.26
SBP (mmHg)	128 (123, 139)	133 (125, 145)	−1.496	0.135
DBP (mmHg)	81.35 ± 13.46	84 (77, 90)	−1.748	0.081
Height (cm)	167 (162, 172)	165 (162, 169.75)	−1.818	0.069
Weight (kg)	106.19 ± 13.56	105.4 (96, 119)	−0.644	0.520
BMI (kg/m^2^)	36.26 (34.17, 41.40)	38.80 (35.66, 42.38)	−2.082	0.037^a^
Waist (cm)	106 ± 9.86	118 (110, 127)		
Hipline (cm)	129.02 ± 10.18	126 (118.25, 133)		
WHR	0.8296 (0.8120, 0.8444)	0.9437 (0.8960, 0.9835)		
GOT (U/L)	22.3 (15.2, 40.3)	29.70 (17.875, 53.55)	−1.608	0.108
GPT (U/L)	16.1 (14.2, 24.7)	19.80 (14.7, 33.775)	−1.689	0.091
GGT (U/L)	27.2 (17.8, 58.5)	29 (19.525, 47.675)	−0.080	0.937
Albumin (g/L)	44.7 (42.2, 45.60)	44.012 ± 3.5266	−0.148	0.883
TG (mmol/L)	1.46 (1.07, 2.74)	1.4650 (1.06, 2.2)	−0.254	0.800
TC (mmol/L)	4.21 (3.70, 5.05)	4.41 (3.96, 5.03)	−1.361	0.173
HDL (mmol/L)	1.08 (0.96, 1.35)	1.07 (0.94, 1.19)	−1.425	0.154
LDL (mmol/L)	2.56 (2.08, 3.26)	2.8 (2.4025, 3.3)	−1.757	0.079
WBC (10^9^/L)	7.06 (6.17, 8.21)	8.0738 ± 2.0911	−2.063	0.039^a^
HB (g/L)	135.3 ± 14.198	135 (127, 143)	−0.271	0.786
PLT (10^9^/L)	285.84 ± 63.563	288 (247.75, 336.75)	−0.849	0.396
HbA1c (%)	5.80 (5.40, 6.60)	5.9 (5.6, 6.575)	−1.141	0.254
FPG (mmol/L)	5.31 (4.83, 6.47)	5.42 (4.92, 6.255)	−0.549	0.583
FINS (μIU/mL)	31.3 (16.99, 45.43)	29.905 (20.685, 45.7625)	−0.669	0.503
C-Peptide (ng/mL)	4.6702 ± 1.61508	4.49 (3.5025, 5.7975)	−0.224	0.823

*Note:* GGT, *γ*-glutamyl transpeptidase; HbA1c, glycosylated hemoglobin A1c; PLT, platelet count.

Abbreviations: BMI, body mass index; DBP, diastolic blood pressure; FINS, fasting insulin; FPG, fasting plasma glucose; GOT, glutamic oxaloacetic transaminase; GPT, glutamic pyruvic transaminase; HB, hemoglobin; HDL, high density lipoprotein; LDL, low density lipoprotein; SBP, systolic blood pressure; TC, total cholesterol; TG, triglycerides; WBC, white blood cells; WHR, waist-to-hip ratio.

^a^ *p* < 0.05.

**Table 3:** Correlation of preoperative clinical indicators between the two groups of males.

Variables	Noncentral obesity	Central obesity	*X* ^2^/*t*/*Z*	*p* value
Number	53	56		
Age (years)	31 (27, 38)	33.86 ± 7.041	−1.454	0.146
SBP (mmHg)	146.75 ± 15.24	146.98 ± 15.775	−0.076	0.939
DBP (mmHg)	90.7 ± 14.027	94.11 ± 14.007	−1.269	0.207
Height (cm)	178 (175, 180)	178.43 ± 6.793	−1.237	0.216
Weight (kg)	144.66 ± 28.23	143.452 ± 25.4925	0.236	0.814
BMI (kg/m^2^)	46.22 ± 8.46	44.96 ± 7.01	0.852	0.396
Waist (cm)	136.189 ± 16.4165	139.384 ± 15.5844		
Hipline (cm)	142 (124, 154.5)	132.73 ± 15.848		
WHR	0.9706 (0.9441, 0.9886)	1.0420 (1.0280, 1.0716)		
GOT (U/L)	38 (22.45, 58.55)	42.30 (24.175, 70.75)	−1.034	0.301
GPT (U/L)	22.2 (16.3, 30.35)	25.9 (15.925, 38.75)	−0.952	0.341
GGT (U/L)	38.1 (26.8, 57.05)	46.35 (35.725, 76.2)	−2.337	0.019^a^
Albumin (g/L)	43.834 ± 3.1714	43.713 ± 2.9123	0.208	0.835
TG (mmol/L)	1.47 (1.125, 2.245)	1.71 (1.3125, 2.52)	−1.419	0.156
TC (mmol/L)	4.57 (4.03, 5.07)	4.85 (4.1025, 5.3)	−1.328	0.184
HDL (mmol/L)	1.06 (0.91, 1.21)	1.025 (0.9225, 1.1425)	−0.825	0.409
LDL (mmol/L)	2.8021 ± 0.86507	3.0414 ± 0.83637	−1.469	0.145
WBC (10^9^/L)	8.2983 ± 2.23721	7.65 (6.8425, 8.385)	−1.055	0.291
HB (g/L)	146.15 ± 16.522	147.21 ± 16.157	−0.034	0.735
PLT (10^9^/L)	283 (231.5, 319)	257.27 ± 55.438	−2.122	0.034^a^
HbA1c (%)	5.70 (5.45, 6.10)	6.25 (5.8, 7.0)	−3.873	< 0.001^c^
FPG (mmol/L)	5.26 (4.9650, 5.7350)	5.81 (4.9725, 6.5475)	−2.258	0.024^a^
FINS (μIU/mL)	36.34 (22.67, 48.615)	35.99 (23.0525, 52.8125)	−0.036	0.971
C-Peptide (ng/mL)	5.02 (4.045, 6.87)	5.005 (3.985, 6.7675)	−0.233	0.815

*Note:* GGT, *γ*-glutamyl transpeptidase; HbA1c, glycosylated hemoglobin A1c; PLT, platelet count.

Abbreviations: BMI, body mass index; DBP, diastolic blood pressure; FINS, fasting insulin; FPG, fasting plasma glucose; GOT, glutamic oxaloacetic transaminase; GPT, glutamic pyruvic transaminase; HB, hemoglobin; HDL, high density lipoprotein; LDL, low density lipoprotein; SBP, systolic blood pressure; TC, total cholesterol; TG, triglycerides; WBC, white blood cells; WHR, waist-to-hip ratio.

^a^ *p* < 0.05.

^c^ *p* < 0.001.

**Table 4:** Differences in weight loss between women with central and noncentral obesity.

	Noncentral obesity	Central obesity	*X* ^2^/*t*/*Z*	*p* value
*Number*	*43*	*208*		
% EWL (1M)	34.94% (28.46%, 39.27%)	28.95% ± 5.77%	−4.614	< 0.001^c^
% TWL (1M)	13.35% ± 3.00%	11.82% ± 2.41%	3.131	0.040^a^

*Number*	*43*	*177*		
% EWL (3M)	59.14% ± 12.95%	53.46% ± 10.11%	2.682	0.01^a^
% TWL (3M)	22.17% ± 3.59%	21.51% ± 3.80%	1.02	0.309

*Number*	*24*	*120*		
% EWL (6M)	78.08% ± 18.15%	77.88% ± 16.27%	0.054	0.957
% TWL (6M)	30.27% ± 6.37%	30.84% ± 5.14%	−0.477	0.634

*Number*	*15*	*72*		
% EWL (12M)	108.97% ± 19.90%	110.67% ± 18.55%	−0.321	0.749
% TWL (12M)	41.94% ± 7.62%	44.79% (41.70%,49.75%)	−1.568	0.117

^a^ *p* < 0.05.

^c^ *p* < 0.001.

**Table 5:** Differences in weight loss between men with central and noncentral obesity.

	Noncentral obesity	Central obesity	*X* ^2^/*t*/*Z*	*p* value
*Number*	*53*	*56*		
%EWL (1M)	28.05% ± 9.44%	29.27% ± 10.79%	−0.629	0.530
%TWL (1M)	12.99% (11.55%, 14.73%)	14.00% ± 2.67%	−1.44	0.150

*Number*	*42*	*50*		
%EWL (3M)	52.00% ± 14.97%	53.30% ± 13.14%	−0.441	0.660
%TWL (3M)	24.06% ± 4.80%	24.46% ± 3.83%	−0.440	0.661

*Number*	*30*	*34*		
%EWL (6M)	68.11% ± 16.32%	68.16% (57.14%, 78.80%)	−0.390	0.696
%TWL (6M)	32.53% ± 4.69%	332.47% ± 4.86%	−0.058	0.954

*Number*	*25*	*24*		
%EWL (12M)	103.50% (97.24%, 107.93%)	97.99% ± 15.50%	−1.460	0.144
%TWL (12M)	48.50% ± 7.37%	48.22% ± 6.64%	0.139	0.890

**Table 6:** Factors influencing percent excess weight loss (%EWL).

	% EWL (1M) *N* = 360		% EWL (3M) *N* = 312		% EWL (6M) *N* = 208		% EWL (12M) *N* = 136	
*Variables*	*Coefficient of correlation B*	*pvalue*	*Coefficient of correlation B*	*pvalue*	*Coefficient of correlation B*	*pvalue*	*Coefficient of correlation B*	*pvalue*
Age	−0.0003	0.586	0.0001	0.854	−0.002	0.159	0.002	0.293
Sex	−0.011	0.371	0.044	0.053	0.027	0.371	0.007	0.861
BMI	−0.006	< 0.001^c^	−0.012	< 0.001^c^	−0.016	< 0.001^c^	−0.014	< 0.001^c^
WHR	−0.147	0.007^b^	−0.029	0.039^a^	−0.058	0.737	−0.087	0.707
Types of obesity	−0.045	< 0.001^c^	−0.038	0.008^b^	0.017	0.544	0.028	0.471
Metabolic syndrome	−0.006	0.561	−0.004	0.787	−0.018	0.490	−0.032	0.350
Hypertension	−0.023	0.012^a^	−0.002	0.862	0.002	0.930	0.017	0.604
T2DM	0.013	0.154	0.019	0.147	0.027	0.220	0.056	0.055
Hyperlipidemia	−0.005	0.526	−0.026	0.035^a^	0.008	0.730	0.001	0.981

Abbreviations: BMI, body mass index; T2DM, type 2 diabetes mellitus; WHR, waist-to-hip ratio.

^a^ *p* < 0.05.

^b^ *p* < 0.01.

^c^ *p* < 0.001.

**Table 7:** Factors influencing percent total weight loss (% TWL).

	TWL (1M) *N* = 360		TWL (3M) *N* = 312		TWL (6M) *N* = 208		TWL (12M) *N* = 136	
*Variables*	*Coefficient of correlation B*	*pvalue*	*Coefficient of correlation B*	*pvalue*	*Coefficient of correlation B*	*pvalue*	*Coefficient of correlation B*	*pvalue*
Age	−0.0002	0.396	0.0001	0.645	−0.001	0.101	0.001	0.464
Sex	−0.002	0.747	0.017	0.084	0.013	0.286	0.015	0.407
BMI	0.0005	0.038^a^	0.001	0.003^b^	0.001	0.010^a^	0.006	< 0.001^c^
WHR	0.076	0.006^b^	−0.005	0.919	−0.013	0.850	−0.041	0.690
Types of obesity	−0.015	0.001^b^	−0.003	0.654	0.009	0.402	0.016	0.359
Metabolic syndrome	−0.001	0.791	0.0001	0.835	−0.003	0.731	−0.006	0.703
Hypertension	−0.01	0.003^b^	−0.001	0.863	0.004	0.684	0.014	0.329
T2DM	0.004	0.274	0.005	0.363	0.009	0.283	0.015	0.236
Hyperlipidemia	−0.002	0.595	−0.012	0.017^a^	0.005	0.593	0.002	0.904

Abbreviations: BMI, body mass index; T2DM, type 2 diabetes mellitus; WHR, waist-to-hip ratio.

^a^ *p* < 0.05.

^b^ *p* < 0.01.

^c^ *p* < 0.001.

## Data Availability

The data that support the findings of this study are available on request from the corresponding author. The data are not publicly available due to privacy or ethical restrictions.
